# Electrochemical detection of aqueous Ag^+^ based on Ag^+^-assisted ligation reaction

**DOI:** 10.1038/srep09161

**Published:** 2015-03-17

**Authors:** Peng Miao, Kun Han, Bidou Wang, Gangyin Luo, Peng Wang, Mingli Chen, Yuguo Tang

**Affiliations:** 1 CAS Key Lab of Bio-Medical Diagnostics, Suzhou Institute of Biomedical Engineering and Technology, Chinese Academy of Sciences, Suzhou 215163, P. R. China; 2 University of Chinese Academy of Sciences, Beijing 100049, P. R. China

## Abstract

In this work, a novel strategy to fabricate a highly sensitive and selective biosensor for the detection of Ag^+^ is proposed. Two DNA probes are designed and modified on a gold electrode surface by gold-sulfur chemistry and hybridization. In the presence of Ag^+^, cytosine-Ag^+^-cytosine composite forms and facilitates the ligation event on the electrode surface, which can block the release of electrochemical signals labeled on one of the two DNA probes during denaturation process. Ag^+^ can be sensitively detected with the detection limit of 0.1 nM, which is much lower than the toxicity level defined by U.S. Environmental Protection Agency. This biosensor can easily distinguish Ag^+^ from other interfering ions and the performances in real water samples are also satisfactory. Moreover, the two DNA probes are designed to contain the recognition sequences of a nicking endonuclease, and the ligated DNA can thus be cleaved at the original site. Therefore, the electrode can be regenerated, which allows the biosensor to be reused for additional tests.

In recent decades, high toxic heavy metal ions, including silver, mercury and lead ions, have brought serious environment and health problems^1^,^2^,^3^. Silver, a widely used element in photographic, medical and electronic industry, is one of the most hazardous metal pollutions^4^,^5^,^6^. Nowadays, large amount of silver waste have been released directly into the environment every year, which may lead to the pollution of ambient water, soil and even food. Finally, silver may accumulate in human body that causes many symptoms and diseases such as skin irritation, argyria, stomach distress, nervous damage, organ edema and even death^7^,^8^,^9^. The mechanism is that the Ag^+^ is able to interact with many biomolecules (e.g., sulfhydryl enzymes) and then alter protein conformations and functions^10^. Therefore, monitoring Ag^+^ levels in aqueous ecosystems especially surface water is of great importance. Conventional analytical methods for Ag^+^ assay in aqueous media include atomic absorption/emission spectroscopy, inductively coupled plasma-mass spectroscopy (ICPMS) and so on^11^,^12^. More recently, a diversity of techniques have also been applied for the analytical purposes, such as voltammetry, chronocoulometry, microscope, fluorescent and colorimetric methods^13^,^14^,^15^,^16^,^17^,^18^,^19^.

In this contribution, we have proposed a novel electrochemical strategy for Ag^+^ assay based on Ag^+^-assisted DNA ligation, denaturation and annealing events of two DNA probes. The biosensor has high sensitivity with the detection limit of 0.1 nM. The selectivity of this biosensor is also excellent. Moreover, the electrode can be regenerated for additional tests by certain treatments. Therefore, the developed biosensor has great potential use for monitoring Ag^+^ in real samples.

## Results and Discussion

**Figure 1:**
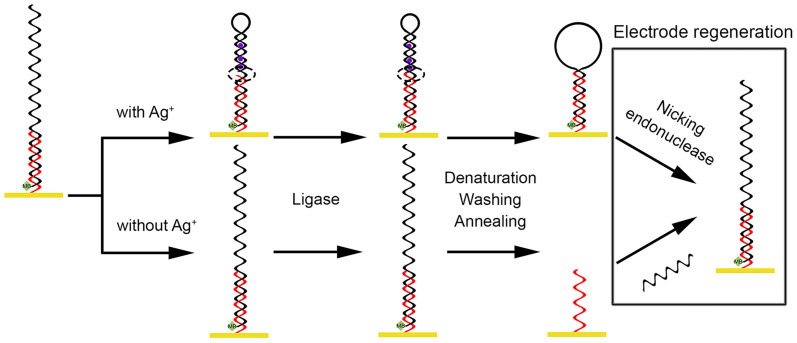
Schematic illustration of the reusable Ag^+^ biosensor.

The principle of the proposed biosensor for the detection of Ag^+^ is depicted in Figure 1. Two oligonucleotides named as capture probe (CP) and signal probe (SP) are employed. Generally, CP is firstly immobilised on the electrode surface via gold-sulfur chemistry^20^. Since CP and SP are designed to contain partial complementary sequences, a hybrid forms on the electrode after the incubation of the electrode in SP solution. However, the 3′ hydroxyl end of SP and the 5′ phosphoryl end of CP are far away from each other. In the presence of the Ag^+^, cytosine-Ag^+^-cytosine composite forms^21^,^22^ and SP can be folded, which facilitates the ligation of the above mentioned hydroxyl and phosphoryl groups catalyzed by T4 DNA ligase^23^. The two DNA probes are thus ligated to be a single probe. Subsequently, denaturation event by heating to 90°C removes unligated SP with methylene blue (MB) from the gold electrode. However, MB molecules on the ligated DNA probe remain on the electrode surface. After an annealing process, it is expected that the ligated DNA probes forms a stem-loop structure and the MB moiety at the 5′ end is able to provide electrochemical signals, which are closely related to the amount of Ag^+^. After one test, the used electrode can be regenerated. Unligated CP hybridizes with added SP to form the hybrid. The ligated DNA probe can be cleaved by a nicking endonuclease at the ligated site. CP and SP modified electrode is thus reproduced.

**Figure 2:**
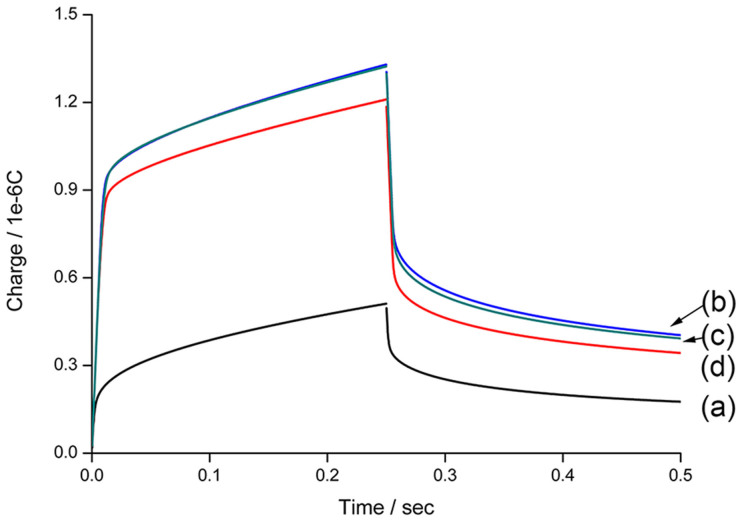
Chronocoulometry curves for the gold electrodes modified with (a) MCH, (b) CP and SP; (c) and (d) are CP and SP modified electrodes with and without the incubation of Ag^+^ before the ligation, denaturation and annealing processes.

Chronocoulometry (CC) is a powerful electrochemical technique, which can provide significant information of hexaammineruthenium(III) chloride ([Ru(NH_3_)_6_]^3+^) bound to DNA probes on the electrode^24^. As shown in Figure 2, after the immobilization of CP and SP on the electrode surface, the charge increases to a great extent. This signal augment is attributed to the attachment of the DNA probes on the electrode surface. After the incubation of the DNA modified electrode with Ag^+^ and the subsequent ligation, denaturation and annealing processes, the CC signal barely changes. The reason is that Ag^+^-mediated conformation change helps the ligation of CP and SP, which are then kept on the electrode during the denaturation. In contrast, an obvious signal drop is observed when the step of Ag^+^ incubation is omitted. It is because ligation reaction cannot occur and SP is released in the solution during denaturation, which leads to fewer [Ru(NH_3_)_6_]^3+^ adsorbed on the electrode surface. The CC results are in accordance to the expectation that Ag^+^ assists the formation of cytosine-Ag^+^-cytosine composite and facilitates the ligation reaction, which determines the amount of remained DNA after denaturation.

**Figure 3:**
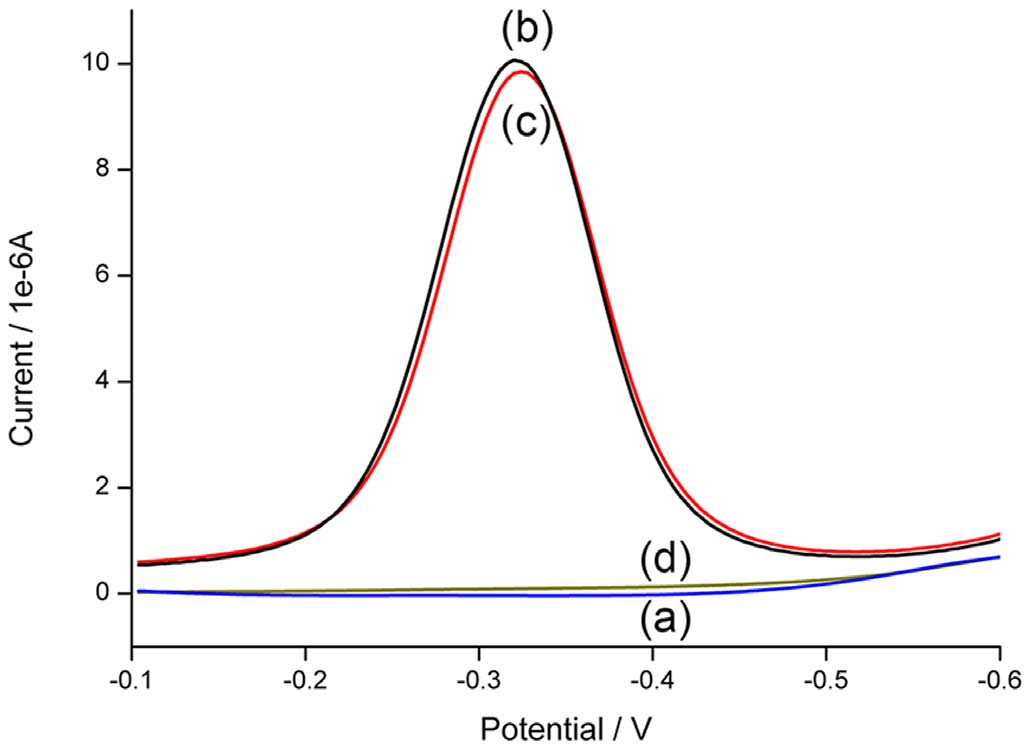
Square wave voltammograms corresponding to (a) bare electrode, (b) CP and SP modified electrode, (c) and (d) are the cases after ligation, denaturation and annealing processes with and without the incubation of Ag^+^.

To further confirm this strategy, square wave voltammetry (SWV) is performed to characterize the steps of the electrochemistry detection method. MB, a thiazine type cationic dye, is covalently modified at the 5′ end of SP, which provides the electrochemical signals^25^. As shown in Figure 3, no peak is observed on the bare electrode. After the modification of CP and SP, MB labeled on SP contributes to a significant peak around −0.32 V. With the help of 100 nM Ag^+^ before denaturation, the decline of SWV peak is negligible, which suggests most of MB moieties are kept on the electrode surface. However, without Ag^+^, a flat SWV curve is exhibited, which supports the fact that a large number of MB are released. The results of CC and SWV have confirmed well the proposed strategy.

**Figure 4:**
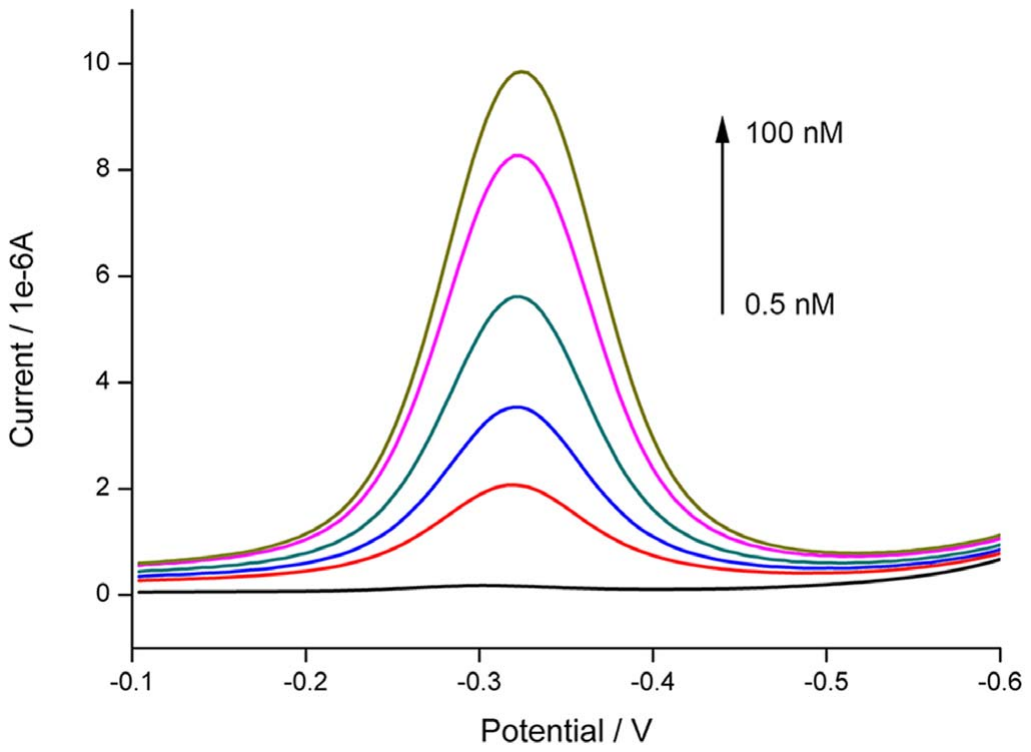
Square wave voltammograms corresponding to CP and SP modified electrodes treated with 0.5, 1, 5, 10, 50, 100 nM Ag^+^ (from bottom to top) before the ligation, denaturation and annealing processes.

**Figure 5:**
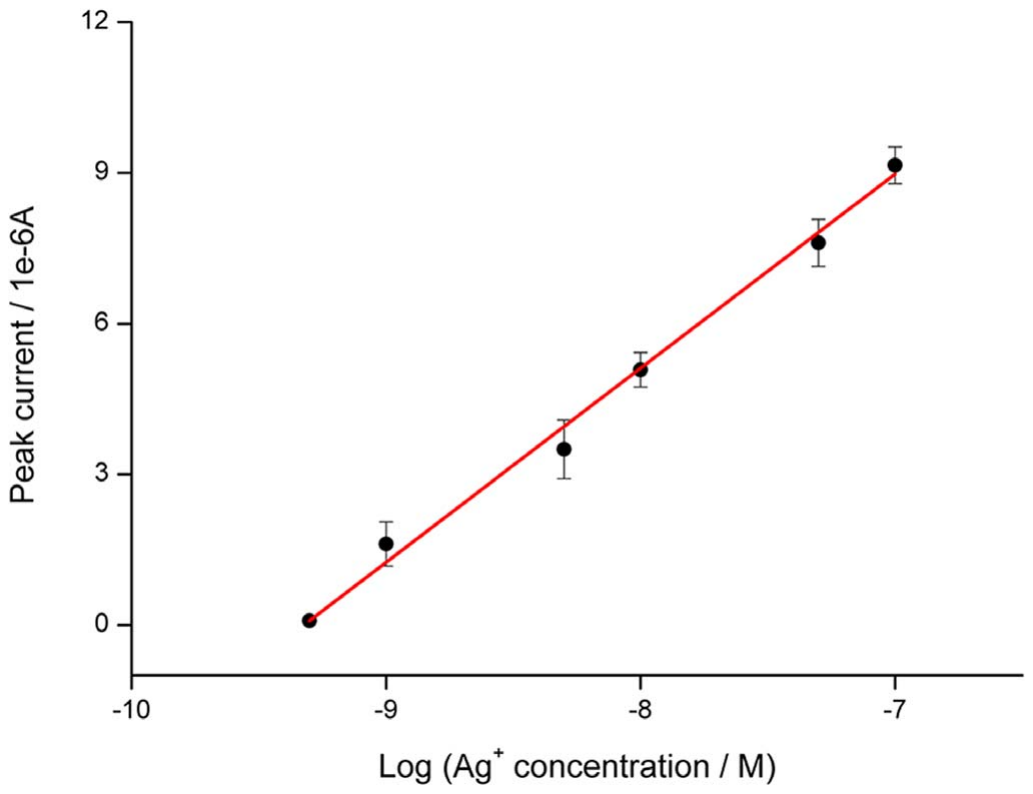
Calibration curve of peak current vs. logarithmic Ag^+^ concentration. Error bars represent standard deviations of three independent measurements.

Ag^+^ with a series of concentrations are prepared and then measured by SWV. As depicted in Figure 4, ligation events and the amount of MB kept on the electrode surface after denaturation are in positive correlation with Ag^+^ concentration. From Figure 5, it is observed that the peak current is linearly related to the logarithmic concentration of Ag^+^. The linear range for Ag^+^ measurement is from 0.5 to 100 nM. The regression equation is *i* = 36.01 + 3.86 log(*c*), in which *i* is the peak current, × 10^−6^ A, and *c* is the concentration of Ag^+^, × M, *n* = 3, *R^2^* = 0.998. The detection limit is calculated to be 0.1 nM (S/N = 3), much lower than the toxicity level of Ag^+^ in drinking water (460 nM) defined by U.S. Environmental Protection Agency (USEPA)^26^.

**Figure 6:**
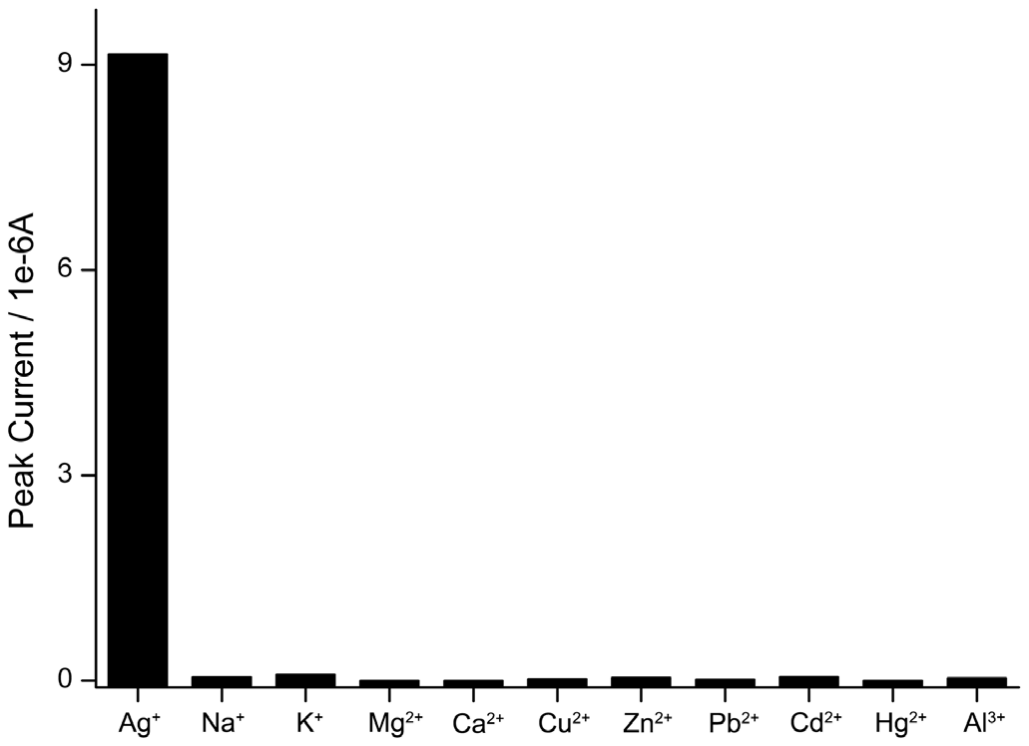
Selectivity of Ag^+^ measurement (100 nM) over other potential interfering ions (1 μM).

The selectivity of this proposed Ag^+^ biosensor is evaluated by introducing some representative interfering ions including Na^+^, K^+^, Mg^2+^, Ca^2+^, Cu^2+^, Zn^2+^, Pb^2+^ Cd^2+^, Hg^2+^, Al^3+^. The peak currents of the above detection cases are compared in Figure 6. It is observed that 100 nM Ag^+^ is able to retain significant intensity of the electrochemical peak, which cannot be achieved by other nonspecific ions with even higher concentrations.

**Figure 7:**
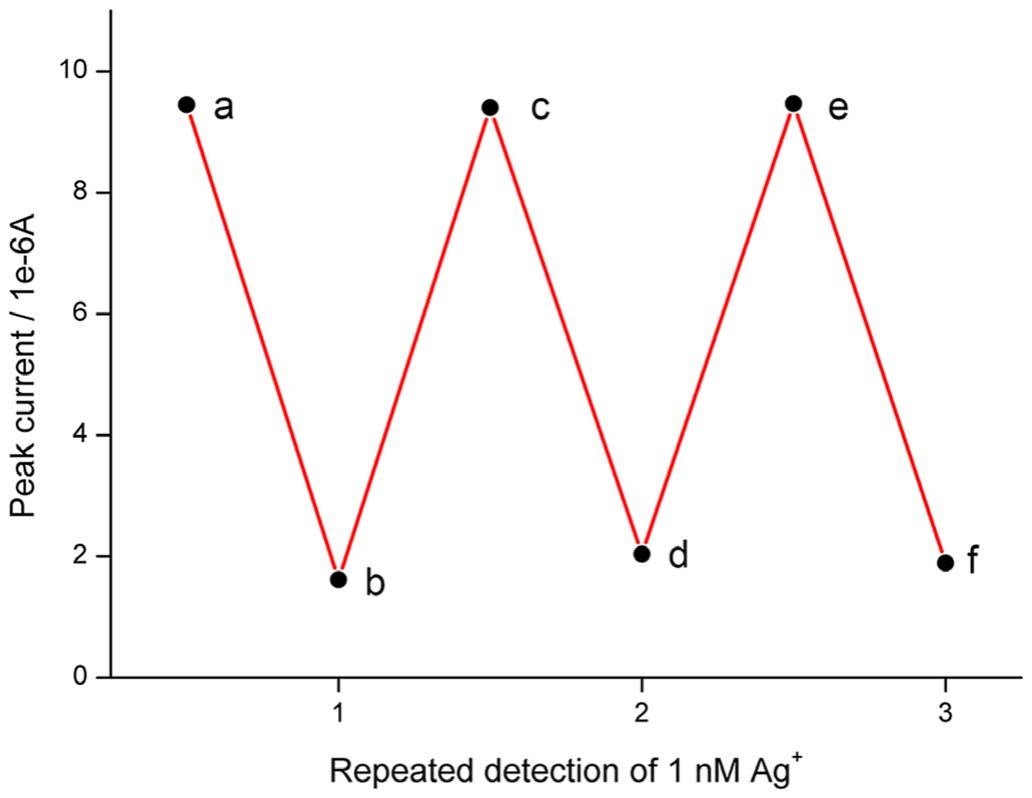
Peak current of the regenerated electrode and the results of the repeated detection of 1 nM Ag^+^.

The proposed biosensor can be reused for additional tests after certain treatments. A two-steps regeneration strategy is raised. The used electrode is firstly incubated in SP solution, which allows the hybridization between single-stranded CP and SP. Then, it is treated by Nt.CviPII to cleave the stem-loop DNA at the original ligated site, opening the loop and regenerating the CP and SP modified electrode. As shown in Figure 7, the reproduced electrode exhibits nearly the same intensity of electrochemical responses as the original CP and SP modified electrode (points a, c, e). The repeated detections of Ag^+^ are also consistent with the primary test (points b, d, f). This unique feature of the biosensor can reduce the cost of Ag^+^ assay, which also makes it possible for future commercialization.

Moreover, practical applications of this method in drinking water and lake water have also been investigated. The samples are tested after filtered by a 0.22 μm membrane. The recoveries and relative errors are satisfactory (Table 1). The results demonstrate this method is well suited to be challenged by real water samples and have great potential use.

In summary, we have proposed the Ag^+^ biosensor based on Ag^+^-assisted ligation reaction with high sensitivity, selectivity and reproducibility. The specific cytosine-Ag^+^-cytosine structure controls the conformational change of the DNA probes on the electrode and facilitates the ligation event, which determines the release of signal probe during denaturation process. The obtained electrochemical response is highly related to the concentration of Ag^+^. The detection limit of this biosensor is as low as 0.1 nM, which is sensitive enough for practical utility. It is also applicable to detect Ag^+^ in drinking water and lake water. More notably, this biosensor is simply operated and regenerated for repeated tests. This ligation reaction-based biosensor can be further modified to detect other analytes by changing the sequences of CP and SP, which can form specific DNA-analyte bioconjugates. Therefore, it holds tremendous potential for cost-effective and sensitive analytical applications.

## Methods

### Materials and chemicals

Nt.CviPII, a kind of nicking endonuclease, and T4 DNA ligase, were purchased from New England Biolabs Ltd. (Beijing, China). Tris(2-carboxyethyl)phosphine hydrochloride (TCEP), [Ru(NH_3_)_6_]^3+^, ethylenediaminetetraacetic acid (EDTA) and mercaptohexanol (MCH) were purchased from Sigma (USA). Silver nitrate was ordered from Nanjing Chemical Reagent Co., Ltd. (Nanjing, China). Other reagents were of analytical reagent grade and used as received. CP and SP were synthesized and purified by Sangon Biotech Co., Ltd. (Shanghai, China). CP was modified with phosphoryl group at the 5′ end and thiol group at the 3′ end. SP was labeled with MB at the 5′ end. The two probes contained partial complementary sequences as follows (in bold). After the hybridization, the underlined sequences listed below could be recognized by Nt.CviPII.

CP: 5′-P-**CCAATTAA**TTTT-(CH_2_)_3_-SH-3′

SP: 5′-MB-**TTAATTGG**TAGCCAACTCATGGGGATCACTTCGCTA-OH-3′

### Preparation of DNA modified gold electrode

The substrate gold electrode was pretreated before any further modification. It was firstly incubated with piranha solution (98% H_2_SO_4_:30% H_2_O_2_ = 3:1) for about 5 min (Caution: piranha solution reacts violently with organic matter!). Then, the electrode was carefully abraded with sand paper and alumina powder of various particle sizes (1.0, 0.3, and 0.05 μm) successively. The electrode was then sonicated in ethanol and water in tandem, each for 5 min. It was incubated with 50% nitric acid for 30 min and then electrochemically cleaned in 0.5 M H_2_SO_4_. The pretreated gold electrode was soaked in 1 μM CP solution for 8 h and then 1 mM MCH for 30 min. MCH acted as the spacer thiol molecules that could make the DNA monolayers well aligned. Afterward, the electrode was incubated with 1 μM SP solution (10 mM phosphate buffer containing 0.25 M NaCl, pH 7.4) for 1 h to form a hybrid on the electrode surface.

### Ag^+^-assisted ligation and further denaturation and annealing

The DNA modified electrode was dipped into sample solutions containing Ag^+^ with different concentrations for 30 min. After that, the electrode was immersed in the ligase reaction buffer containing 5 unit mL^−1^ T4 DNA ligase at 22°C for 1 h. The electrode was then rinsed with Tris-HCl buffer (pH 7.5) with 0.5% Tween and then pure water. Subsequently, the electrode was incubated in water at 90°C for 5 min and washed for three times. Then, the electrode was slowly cooled down to room temperature.

### Electrochemical measurements

CHI660D was used as the electrochemical analyze for all electrochemical experiments. Three electrode system was employed, which consisted of a saturated calomel reference electrode (SCE), a platinum wire counter electrode and working gold electrode (gold area: 3.142 mm^2^). Although it is reported that platinum counter electrode may dissolve into electrolyte^27^, the resulted ions do not bind to DNA bases and may not influence DNA engineering processes in the experiments. CC was carried out in 10 mM Tris-HCl with 50 μM [Ru(NH_3_)_6_]^3+^ (pH 7.4). The pulse period was set to be 250 ms. SWV was performed in 20 mM Tris-HCl (pH 7.5). The amplitude was 25 mV, the step potential was 4 mV and the frequency was 90 Hz.

## Author Contributions

P.M., B.D.W. and Y.G.T. designed the experiments. P.M. and K.H. conducted the experiments. G.Y.L., P.W. and M.L.C. participated in the experiments and discussions. P.M. wrote the paper.

## Figures and Tables

**Table 1:** Measurements of Ag^+^ levels in drinking water and lake water

Samples	Added (nM)	Detected (nM)	Recovery (%)	Relative error (%)
Drinking water	10	12.15	121.50	5.11
	30	31.03	103.43	3.48
	50	48.69	97.38	4.79
Taihu Lake water	10	11.22	112.20	3.77
	30	28.61	95.37	4.24
	50	53.14	106.28	1.58
